# Childhood adversity and common mental disorders in young employees in Sweden: is the association affected by early adulthood occupational class?

**DOI:** 10.1007/s00127-020-01874-0

**Published:** 2020-05-13

**Authors:** Emma Björkenstam, Magnus Helgesson, Ellenor Mittendorfer-Rutz

**Affiliations:** 1 Department of Clinical Neuroscience, Division of Insurance Medicine, Karolinska Institutet, 171 77 Stockholm, Sweden; 2 Department of Community Health Sciences, Fielding School of Public Health and California Center for Population Research, University of California Los Angeles, Los Angeles, CA USA; 3 Department of Neuroscience, Psychiatry, Uppsala University, Uppsala, Sweden

**Keywords:** Childhood adversity, Cohort, Sweden, Epidemiology, Occupational class, Common mental disorder, Employment, Occupation, Young adults

## Abstract

**Background:**

Childhood adversities are associated with an elevated risk for common mental disorders (CMDs). Whether the strength of the association also holds for young employees is unclear. Given the increase in CMD rates in young adults over the past decade, identification of risk factors has important implications for future public health interventions. The current study aimed to investigate the effects of childhood adversities on CMDs. Additionally, the role of occupational class (non-manual/manual workers) in the relationship was examined.

**Methods:**

This population-based longitudinal cohort study included 544,003 employees, 19–29 years, residing in Sweden in 2009. Adversities included parental death, parental mental and somatic disorders, parental separation or single-parent household, household public assistance and residential instability. Estimates of risk of CMDs, measured as prescription of antidepressants and/or psychiatric care with a clinical diagnosis of CMDs, between 2010 and 2016 were calculated as relative risks (RR) with 95% confidence intervals (CI), using a modified Poisson regression analysis. Occupational class (non-manual/manual workers) was explored as a potential moderator.

**Results:**

In both manual and non-manual workers, childhood adversities were associated with an elevated risk of subsequent CMDs. The risk was moderated by occupational class, i.e., especially pronounced risk was found in manual workers who had experienced cumulative adversity (adjusted RR 1.76, 95% CI 1.70–1.83) when compared to non-manual workers with no adversity. Among the adversities examined, having had a parent treated for a mental disorder, having grown up in a household living on public assistance or having experienced residential instability were the strongest predictors of CMDs.

**Conclusion:**

Our findings suggest that, among young employees, manual workers with a history of multiple childhood adversities are especially vulnerable to subsequent CMDs.

**Electronic supplementary material:**

The online version of this article (10.1007/s00127-020-01874-0) contains supplementary material, which is available to authorized users.

## Introduction

Common mental disorders (CMDs), i.e., depressive, anxiety and stress-related disorders, in young adults have increased in many Western countries over the past decade [1–3]. Recent statistics from the World Health Organization (WHO) showed that CMDs account for the largest proportion of mental disorders in children and adolescents [4]. According to their report, almost 20% of the population in the WHO European Region aged 10–19 years have a mental disorder, of which CMDs accounts for over 40% [4]. Given this increase in CMD rates over the past decade, identification of risk factors has important implications for future public health interventions. The first onset of CMDs often occurs in childhood or adolescence [5]. For this reason, studies on pathways to CMDs in young individuals are of essential importance to prevent the health and social impairment, CMDs in young age imply.

Substantial research has pointed out childhood adversity as particularly detrimental to CMDs [6–9]. Childhood adversities that have been linked to CMDs include parental separation, single parenthood, parental criminality, parental psychiatric and severe chronic somatic morbidity [6, 9, 10].

Studies have further shown that CMDs occur more frequently among persons in socioeconomically disadvantaged groups [11], and that the socioeconomic gradient in CMDs is evident already in childhood. Moreover, individuals with a history of childhood adversity have been shown to have an elevated risk for low educational attainment [12–14] and work disability in terms of unemployment [13, 15–17], sickness absence (SA) [13, 18] and disability pension (DP) [13, 18, 19], which in turn are associated with CMDs [20]. These associations go both ways, as CMDs also may have a strong effect on the individuals work ability [21, 22]. For these reasons, analyses on the adversity–CMD link should be adjusted for measures of work disability in young adulthood.

Although the known associations between childhood adversity and CMDs in the general population are substantial, whether these associations can be extended to young adults who are in the labor market is less known. Given that employees in general constitute a group in better health than the general population [23], one hypothesis not yet tested is if the negative effects of childhood adversity would be also present in such a group. Young adults have their entire working life in front of them. For this reason, identification of risk factors for CMDs in young adults in working life has important implications for the prevention of early labor market exclusion due to mental disorders.

As childhood adversities often lead to low educational attainment and unemployment, there may further be differences in CMD risk due to occupational class, often categorized as manual- and non-manual workers. Here, epidemiological studies on socioeconomic inequalities in CMDs have consistently shown that manual workers, including workers within service and sales occupations, have an elevated risk for CMDs compared to non-manual workers, such as those working in professional occupations (e.g., sales professionals and accountants) [11, 24]. Studies have shown that other determinants of socioeconomic status (SES) such as educational level have an influence on the adversity–CMD relationship. Since occupational class is associated with both childhood adversity and CMDs, it may play an important moderating role in this association, but to date studies examining this are lacking. A limited number of studies have shown that a combination of CA and low SES in adulthood is associated with several behavioral risk factors of CMD including obesity and physical inactivity [25] and one may, therefore, expect that a combination of the two lead to an elevated risk of CMD, yet this has not been studied to date. Here, one possible pathway is that exposure to adversity early in life decreases the probability of obtaining a higher education, that in turn leads to a higher probability of working in manual occupations, that may be characterized by adverse psychosocial work environments, also associated with CMDs.

To address this gap in the literature, the current study used a large cohort of nearly 550,000 young employed individuals in Sweden, born between 1980 and 1990, to investigate associations between childhood adversity and CMDs. We further examined whether there were differences in non-manual and manual workers in the childhood adversity–CMD relationship. More specifically, the following research questions were examined:

1. Is there an association between specific and cumulative childhood adversity, respectively, and risk of subsequent CMDs in young employees?
2. Do associations between childhood adversity and subsequent CMDs differ between non-manual and manual employees?

## Material and methods

We used the unique (de-identified) Swedish personal identity number to link information from several population-based health care and administrative registers [26]:

The Longitudinal Integration Database for Health Insurance and Labor Market Studies (LISA) register contains data from the labor market and from the educational and social sectors [27]. The National Patient Register (NPR) includes information on inpatient care since 1987 and for specialized outpatient care since 2001 with almost complete coverage [28]. Diagnoses in NPR are coded according to the International Classification of Diseases version 10 (ICD-10) [29]. The Prescribed Drug Register (PDR) contains patient identities for all dispensed prescribed drugs to the entire Swedish population since July 2005. Pharmaceuticals in PDR are grouped according to the Anatomical Therapeutic Chemical Classification System (ATC). The Cause of Death Register (CDR) comprises information on all deaths of Swedish residents since 1952. The validity of this register is high, and the cause of death is missing in < 1% of the deceased [30]. Families were linked together through the Multi-Generation Register, which contains all known relationships between children and parents (born 1932 or later) since 1961. Finally, the Total Population Register includes information on age, sex, place of residence and other demographic characteristics.

### Study population

Included were all individuals born in Sweden, aged 19–29 years, residing in the country on December 31st, 2009 (*n* = 1,074,160). Only those classified as being in employment in the register, and for whom parental information was available were included (*n* = 669,179). After excluding those with incomplete or missing information on occupational class (*n* = 77,683) and those treated for CMD in the period 2006–2009 (*n* = 47,493), the final study sample included 544,003 individuals.

### Exposure

A total of six childhood adversities that were available in the registers were included in the study (see Supplementary Table 1 for definition): parental death, parental mental disorder, parental somatic disorder, parental separation or single-parent household, household public assistance and residential instability. All these adversities have been linked to significant adverse health and social outcomes [6, 19]. Based on data availability, the first three adversities were measured between birth and age 18 years; whereas, the remaining adversities were captured from 1985 or 1990 and onwards (Supplementary Table 1). To assess cumulative exposure to the studied adversities, the total number was summed and grouped into 0, 1, 2, and 3 or more adversities. Each adversity was weighted equivalently in the analyses.

**Table 1:** Selected cohort characteristics, by exposure to childhood adversity, in employed individuals aged 19–29 years residing in Sweden in 2009

	All	No childhood adversity	Parental death	Parental mental disorder	Parental somatic disorder	Parental separation or single-parent household	Household public assistance	Residential instability	Total number of adversities		
									1	2	3 +
All (n, row percent)	544,003 (100)	294,808 (54)	5,539 (1)	40,284 (7)	66,558 (12)	201,148 (37)	18,179 (3)	20,173 (4)	171,909 (32)	55,995 (10)	21,291 (4)
Sociodemographic factors^a^											
Sex											
Women	261,042 (48)	140,081 (48)	2,629 (47)	19,307 (48)	31,782 (48)	98,154 (49)	9,000 (50)	10,253 (51)	83,148 (48)	27,476 (49)	10,337 (49)
Men	282,961 (52)	154,727 (52)	2,910 (53)	20,977 (52)	34,776 (52)	102,994 (51)	9,179 (50)	9,920 (49)	88,761 (52)	28,519 (51)	10,954 (51)
Education (years)											
Compulsory school (< 9)	35,481 (7)	11,782 (4)	512 (9)	4,826 (12)	5,170 (8)	20,909 (10)	3,453 (19)	3,060 (15)	13,613 (8)	6,738 (12)	3,348 (16)
High school (10–12)	338,198 (62)	175,576 (60)	3,491 (63)	26,379 (65)	41,257 (62)	133,971 (67)	12,140 (67)	12,760 (63)	111,954 (65)	36,700 (66)	13,968 (66)
College or university (> 12)	169,370 (31)	107,093 (36)	1,520 (27)	8,968 (22)	20,003 (30)	45,748 (23)	2,478 (14)	4,258 (21)	46,009 (27)	12,392 (22)	3,876 (18)
Missing	954 (0)	357 (0)	16 (0)	111 (0)	128 (0)	520 (0)	108 (1)	95 (0)	333 (0)	165 (0)	99 (0)
Occupational class											
Manual workers	394,954 (73)	202,301 (69)	4,177 (75)	32,070 (80)	48,811 (73)	158,792 (79)	15,570 (86)	16,454 (82)	130,530 (76)	44,560 (80)	17,563 (82)
Non-manual workers	149,049 (27)	92,507 (31)	1,362 (25)	8,214 (20)	17,747 (27)	42,356 (21)	2,609 (14)	3,719 (18)	41,379 (24)	11,435 (20)	3,728 (18)
Health-related factors^b^											
Somatic morbidity^c^	287,976 (53)	151,571 (51)	2,918 (53)	22,783 (57)	39,094 (59)	110,825 (55)	10,555 (58)	11,452 (57)	92,844 (54)	31,338 (56)	12,223 (57)
Work disability factors^a^											
No long-term sickness absence (LTSA)	541,152 (99.5)	293,449 (99.5)	5,510 (99.5)	39,992 (99.3)	66,147 (99.4)	199,932 (99.4)	18,023 (99.1)	20,023 (99.3)	170,964 (99.5)	55,619 (99.3)	21,120 (99.2)
LTSA (> 90 days/year)	2,851 (0.5)	1,359 (0.5)	29 (0.5)	292 (0.7)	411 (0.6)	1,216 (0.6)	156 (0.9)	150 (0.7)	945 (0.5)	376 (0.7)	171 (0.8)
No disability pension (DP)	543,151 (99.8)	294,380 (99.9)	5,531 (99.9)	40,184 (99.8)	66,448 (99.8)	200,800 (99.8)	18,136 (99.8)	20,120 (99.7)	171,644 (99.8)	55,896 (99.8)	21,231 (99.7)
DP	852 (0.2)	428 (0.1)	8 (0.1)	100 (0.2)	110 (0.2)	348 (0.2)	43 (0.2)	53 (0.3)	265 (0.2)	99 (0.2)	60 (0.3)

^a^In 2009

^b^In 2006–2009

^c^Defined by ATC-codes A10, N03A excluding mood stabilizers; and/or any ICD-10 code, excluding codes F00–99, O80 and R00–99

### Moderator

Occupational class, obtained from the LISA register in 2009, was coded according to the Swedish Standard Classification of Occupations [31]. The SSYK system has 10 categories which were dichotomized into manual and non-manual workers following a simplified version of the classification by Thell [32], which is based on what the work entails, education required, and supervision responsibility. The non-manual workers consist of legislators, senior officials, and managers; professionals, technicians and associate professionals; and clerks. The manual workers include service workers and shop sales workers; skilled agricultural and fishery workers; craft and related trades workers; plant and machine operators and assemblers; and elementary occupations.

### Outcome

CMDs were defined as having a main diagnosis for CMDs, including major depressive disorders (ICD-10: F32–33), phobic anxiety disorders (ICD-10: F40), other anxiety disorders (ICD-10: F41), obsessive–compulsive disorders (ICD-10: F42) and reaction to severe stress, and adjustment disorders (ICD-10: F43), in inpatient care or specialized outpatient care, or being prescribed antidepressants (ATC: N06A) during the follow-up period (i.e., January 1st 2010–December 31st 2016). We conducted sensitivity analysis separating diagnosis-based and medication-based outcome, respectively.

### Covariates

A range of potential confounders, with known associations to both childhood adversity and CMDs, were included in the analyses. If not stated otherwise, all confounders were measured in the year 2009. Adjustments were made for age and sex. Parental country of birth was categorized as Sweden (both parents Swedish-born), mixed (one Swedish-born), other Nordic (at least one parent born in Denmark, Finland, Norway or Iceland), other European Union and non-European Union (at least one parent). Adjustments were further made for education, family situation and type of residential area. Long-term sickness absence (LTSA) (> 90 net days per year), and disability pension (DP) were used as indicators of work disability. Adjustments were also made for somatic morbidity in 2006–2009, defined as inpatient or specialized outpatient care with a main diagnosis for somatic disease, or utilization of certain prescribed medications. For diagnoses, all ICD-10 codes were considered, with the exception of codes related to mental disorder (ICD-10: F00–F99), codes related to pregnancy and childbirth (i.e., ICD-10: O80), and symptoms, signs and abnormal clinical and laboratory findings (ICD-10: R00–R99). For prescribed medication use, the following drugs were considered: antidiabetics (ATC: A10), antiepileptic medication (ATC: N03A, excluding mood stabilizers). Missing values in any confounder were grouped as separate categories in multivariate analyses.

### Statistical analysis

Statistical analyses were conducted using SAS, v.9.4 and Stata IC v 13. We conducted modified robust Poisson regression models, to estimate the relative risk of CMDs associated with CAs and occupational status [33]. Results are presented as relative risks (RR) with 95% confidence intervals (CIs). In the regression models, occupational class was treated as non-time-varying binary data. In the main analysis, one crude and one adjusted regression models were analyzed: Model 2 adjusted for age, sex, education, family situation, type of residential area, work disability factors (LTSA and DP) and somatic morbidity. The reference group comprised non-manual workers with no childhood adversity.

We conducted a sensitivity analysis, in which we also included the 47,493 individuals with a history of CMDs (i.e., in a cohort comprising both incident and recurrent cases of CMDs).

## Results

Table 1 shows that nearly half (46%) of the individuals had experienced one or more adversities, of which the most common was parental separation or growing up in a single-parent household (37%). Compared with children without any adversities, those exposed to at least one adversity were more likely to have a lower level of education, to have parents who were born outside of Sweden and to live in big city areas (Table 1 and Supplementary Table 2). Among those who had experienced three or more adversities, 82% were manual workers (compared to 69% of those with no adversity).

Table 2 presents the crude and adjusted RRs with 95% CIs for CMDs by occupational class and exposure to childhood adversity. Regardless of childhood adversity exposure, manual employees had a higher CMD risk when compared to non-manual employees (crude RR 1.27; 95% CI 1.25–1.29). Among both non-manual and manual employees, all adversities were associated with elevated risk for CMDs. The highest RR was observed for manual employees who grew up in households receiving public assistance (crude RR 1.89; 95% CI 1.83–1.95). The second and third highest RRs were observed for parental mental disorder (crude RR 1.87; 95% CI 1.83–1.92) and residential instability (crude RR 1.83; 95% CI 1.77–1.89). When sociodemographics, work disability factors and somatic morbidity were added to the initial model, all RRs decreased slightly (Table 2, Model 2).

**Table 2:** Relative risks (RR) with 95% confidence intervals (CI) for associations between childhood adversity, occupational class, and common mental disorders (CMDs) in employees in Sweden, aged 19–29 years residing in Sweden in 2009^a^

Childhood adversity	*n* CMD (%)			Model 1^a^		Model 2^b^	
	All	Non-manual workers	Manual workers	Non-manual workers	Manual workers	Non-manual workers	Manual workers
All	73,783 (14)	16,896 (11)	56,887 (14)	1 (REF)	1.27 (1.25–1.29)	1 (REF)	1.17 (1.14–1.19)
Parental death							
No	72,898 (14)	16,725 (11)	56,173 (14)	1 (REF)	1.27 (1.25–1.29)	1 (REF)	1.17 (1.14–1.19)
Yes	885 (16)	171 (13)	714 (17)	1.11 (0.96–1.28)	1.51 (1.41–1.62)	1.09 (0.95–1.26)	1.32 (1.23–1.41)
Parental mental disorder							
No	65,799 (13)	15,547 (11)	50,252 (14)	1 (REF)	1.25 (1.23–1.28)	1 (REF)	1.16 (1.14–1.19)
Yes	7,984 (20)	1,349 (16)	6,635 (21)	1.49 (1.41–1.57)	1.87 (1.83–1.92)	1.42 (1.35–1.49)	1.61 (1.56–1.65)
Parental somatic disorder							
No	63,884 (13)	14,571 (11)	49,133 (14)	1 (REF)	1.26 (1.24–1.29)	1 (REF)	1.16 (1.14–1.18)
Yes	9,899 (15)	2,145 (12)	7,754 (16)	1.08 (1.03–1.12)	1.41 (1.38–1.45)	1.06 (1.02–1.11)	1.27 (1.24–1.31)
Parental separation or single-parent household							
No	40,426 (12)	11,059 (10)	29,367 (12)	1 (REF)	1.20 (1.18–1.22)	1 (REF)	1.15 (1.12–1.17)
Yes	33,357 (17)	5,837 (14)	27,520 (17)	1.33 (1.29–1.37)	1.67 (1.64–1.71)	1.26 (1.22–1.3)	1.47 (1.43–1.50)
Household public assistance							
No	70,027 (13)	16,443 (11)	53,584 (14)	1 (REF)	1.26 (1.24–1.28)	1 (REF)	1.17 (1.14–1.19)
Yes	3,756 (21)	453 (17)	3,303 (21)	1.55 (1.42–1.68)	1.89 (1.83–1.95)	1.37 (1.26–1.49)	1.51 (1.46–1.57)
Residential instability							
No	69,840 (13)	16,329 (11)	53,511 (14)	1 (REF)	1.26 (1.24–1.28)	1 (REF)	1.16 (1.14–1.19)
Yes	3,943 (20)	567 (15)	3,376 (21)	1.36 (1.26–1.47)	1.83 (1.77–1.89)	1.26 (1.17–1.36)	1.50 (1.44–1.55)

^a^Each reference group comprises non-manual workers without the adversity

^b^Model 1: Crude

^c^Model 2: Adjusted for age, sex, education, family situation, parental country of birth, residential area, LTSA and DP in 2009, and somatic morbidity

In a graded manner, cumulative exposure childhood adversity was associated with higher risks for CMDs for both non-manual and manual employees (Table 3). Compared to non-manual employees with no adversity, individuals with 3 + adversities had over a twofold elevated risk for CMDs (crude RR: 2.12; 95% CI 2.05–2.19). Part of the elevated risk was explained by the confounders (Table 3, Model 2); the RR decreased to 1.76 (95% CI 1.70–1.83). Corresponding RR among non-manual employees with 3 + adversities was 1.51 (95% CI 1.40–1.63) after adjusting for all covariates.

**Table 3:** Associations between cumulative childhood adversity, occupational class, and common mental disorders (CMDs) in employees in Sweden, aged 19–29 years residing in Sweden in 2009

Total number of childhood adversities	*n* CMD (%)			Model 1^a^		Model 2^b^	
	All	Non-manual workers	Manual workers	Non-manual workers	Manual workers	Non-manual workers	Manual workers
0	33,896 (11)	9,465 (10)	24,431 (12)	1 (REF)	1.18 (1.15–1.21)	1 (REF)	1.13 (1.1–1.16)
1	25,354 (15)	5,085 (12)	20,269 (16)	1.20 (1.16–1.24)	1.52 (1.48–1.55)	1.16 (1.13–1.20)	1.37 (1.34–1.41)
2	10,102 (18)	1,721 (15)	8,381 (19)	1.47 (1.40–1.54)	1.84 (1.79–1.89)	1.38 (1.32–1.45)	1.59 (1.54–1.64)
3 +	4,431 (21)	625 (17)	3,806 (22)	1.64 (1.52–1.76)	2.12 (2.05–2.19)	1.51 (1.40–1.63)	1.76 (1.70–1.83)

Relative risks (RRs) with 95% confidence intervals (CIs)

^a^Model 1: Crude

^b^Model 2: Adjusted for age, sex, education, family situation, parental country of birth, residential area, LTSA and DP in 2009 and somatic morbidity

The sensitivity analysis in which we separated diagnosis-based and medication-based outcome, respectively, revealed similar results as those presented in the main analysis (data not shown).

Last, in the sensitivity analyses where we included the 47,493 individuals with a history of CMDs (Supplementary Tables 3–4), the risks were similar to the main analyses.

## Discussion

### Main findings

In this register-based national cohort study of 544,003 young employees in Sweden, we examined the associations between childhood adversity, early adulthood occupational class and subsequent CMDs. Our findings indicate that manual employees more often have a history of cumulative adversity compared to non-manual workers. Moreover, manual workers with a history of childhood adversity have a higher risk of subsequent CMDs, particularly those with cumulative adversity, when compared with non-manual workers without adversity. Among the adversities examined, having had a parent treated for a mental disorder, having grown up in a household living on public assistance or having experienced residential instability were the strongest predictors of CMDs.

### Study findings in relation to prior research

Our findings revealed that exposure to childhood adversity was more common in manual workers compared with non-manual workers. For example, among manual workers, 50% had experienced one or more adversities compared to 38% of non-manual workers. Earlier studies on childhood adversity and occupational class, most often based on self-reported survey data, have typically looked at unemployment as a measure of occupation-based SES rather than specific occupational classes, showing that childhood adversity is associated with an increased risk of unemployment [13, 16, 17]. Using education as an individual-based SES, studies have reported that increased childhood adversity is associated with the decreased prevalence of higher education [13, 15, 17]. Similar findings were also seen in our study, where those exposed to 3 + adversities to a lesser extent had a college degree (18% vs. 36% in those with no CA).

Our findings indicated a strong positive association between multiple types of childhood adversity and subsequent CMDs in both manual and non-manual workers. Prior studies in more general settings, i.e., not exclusively in employees, have demonstrated that childhood adversities, such as the ones used in our study, predict later CMDs [6–9]. Among the different adversities, parental mental disorder tended to be the strongest predictor in all regression models. It is important to keep in mind that there may be a genetic component associated with this specific adversity, such that individuals whose parental have been treated for a mental disorder have higher CMD risk themselves. Furthermore, growing up in a single-parent household or in a household living on public assistance were strong predictors of CMDs. These findings are consistent with earlier studies [34]. For example, a large-scale Swedish study examining mortality and morbidity in children with single parents showed that, although growing up in a single-parent family often is associated with a wide range of other social adversities including lack of household resources, the increased risk of mental health problems for children of single parents remained even after taking other adversities into account [35]. The associations found in our study remained significant even after adjusting for important work-related factors, including LTSA and DP. Such important factors have not been taken into account in prior studies. The present study further demonstrated that the CMD risk grew higher with increasing number of adversities. A similar dose–response pattern has been shown in prior studies [6–8].

Various pathways through which childhood adversity influences CMDs have been discussed including both biological and psychosocial mechanisms [36, 37]. Exposure to childhood adversity is a major contributor to increased stress levels in childhood [36], and biological explanations have suggested that childhood adversities contribute to stress-induced brain dysfunction that in turn may lead to mental health problems, including CMDs [36, 37]. Psychological explanations on the other hand suggest that childhood adversity may lead to emotional dysfunction, and that children with poor emotional competence may have higher likelihood of CMDs [36, 38, 39].

Our findings shed further light on the mechanisms underlying the link between childhood adversity and CMDs by examining the role of occupational class. In line with prior studies in various settings [11, 34], we found a socioeconomic gradient in this young cohort, such that manual-workers, regardless of childhood adversity exposure, had significantly elevated risk for CMDs compared to non-manual workers. This inverse association between SES and CMD has been well documented, showing that a socioeconomic gradient is evident already in childhood [11]. Here, different explanations have been proposed. For example, a life course approach suggests that people from socioeconomically disadvantaged backgrounds are more likely to accumulate risk throughout life that those in higher SES groups [40]. Others have proposed that loss of control over one’s life, which is more common in socioeconomically disadvantaged groups, may contribute to socioeconomic inequalities in CMD [11]. Our study showed that the risk was particularly pronounced in young manual workers with a history of childhood adversity. Similar to our findings, one recent Finnish study on childhood adversity, adult occupation-based SES and work disability, in which low SES was defined as manual worker occupations, found that those with a history of reported adversity and low SES had a higher risk of disability due to mental disorders [18]. Although that study focused on the employed population, the study participants were older than those in our study.

Since childhood adversity has been associated with less favorable educational outcomes, one possible pathway is that exposure to adversity early in life decreases the probability of obtaining a higher education. As a consequence, these individuals have a higher risk to later work in manual occupations that may be characterized by an adverse psychosocial work environment. Here, studies have shown different indicators of an adverse psychosocial work environment to be associated with CMDs [41–43]. This has been especially evident when it comes to job strain, i.e., having high job demands with little possibility to control the work situation and to get rewarded for the efforts.

Exposure to childhood adversity appears to be common; nearly half of the individuals included in our study had experienced at least one adversity and 14% had a history of two or more adversities. Our findings give further support to the research underlining the importance of the childhood environment for mental health later in life. Given that experience of childhood adversity is common, early and efficient support of disadvantaged children is of utter importance for improving their long-term mental health. Several studies have stressed the importance of preventive interventions [44, 45]. Moreover, policies and interventions to reduce CMDs need to consider the social background as an important risk or protective factor.

### Strengths and limitations

This study has several strengths including the longitudinal, population-based design and use of registers of high quality and completeness. Previous studies on childhood adversity and CMDs have often been retrospective and based on self-reported information, entailing risk for incorrect answers due to, e.g., poor recall or blocking of certain memories [46]. Furthermore, the large population size should be interpreted in the context of the following limitations: The range of adversities is far from exhaustive and we do not assess the severity, duration or sequencing of any of these adversities. Another limitation is that we have not examined the fluidity of childhood adversities but rather, as done by others, treated them as discrete life events, and all adversities are weighted equality important to CMDs. However, both the consistency of our results with other studies and the large cohort with high-quality data lend confidence to the validity of our findings. Since we are using register-based diagnoses and retrieved prescriptions for antidepressants to capture CMDs, we have likely misclassified milder cases as well as undiagnosed and/or untreated individuals as free of disease. Also, prescription of antidepressants was used as proxy for CMDs, and even though most prescribing of antidepressants is for CMDs, these drugs may also be used for other indications [47]. The lack of data on occupational class throughout the entire follow-up period might have led to over- or underestimation of the reported estimates. Finally, this is an observational study with obvious limitations regarding causality in the relationship between childhood adversities and mental disorders.

## Conclusion

In conclusion, this study extends earlier research by providing evidence of associations between childhood adversities and CMDs also in a population of young adults in employment. Our findings further suggest that, among young employees, manual workers with a history of multiple childhood adversities are especially vulnerable to subsequent CMDs. These findings should be taken into account in the attempt to reduce CMDs in the young working population.

## Electronic supplementary material

Below is the link to the electronic supplementary material.

Supplementary file1 (DOCX 25 kb)
