# Supplementary material for: Childhood adversity and common mental disorders in young employees in Sweden: is the association affected by early adulthood occupational class?
Source: Soc Psychiatry Psychiatr Epidemiol. 2020 May 13;56(2):237–46. doi: 10.1007/s00127-020-01874-0 (PMC7870617; doi:10.1007/s00127-020-01874-0)
Supplement: Supplementary file 1 — Supplementary file1 (DOCX 25 kb) [file 127_2020_1874_MOESM1_ESM.docx]

***Supplementary Table 1.*** *Definitions and classification of childhood adversity*

| **Childhood adversity** | **Definition** | **ICD Classification** | **Data Source (years)** |
| --- | --- | --- | --- |
| Parental death | Parental death | N/A | Causes of Death Register (1980-2008) |
| Parental mental disorder | Parental hospitalization for mental disorder | ICD-9: 290-319  ICD-10: F00-F99 | National Patient Register (1980-2008) |
| Parental somatic disorder | Parental hospitalization for any of the following conditions [48]: myocardial infarction, congestive heart failure, peripheral vascular disease, cerebrovascular disease, dementia, chronic pulmonary disease, rheumatic disease, liver disease, diabetes mellitus, hemiplegia/paraplegia, renal disease, malignancy, metastatic tumors, and AIDS/HIV. | See Brusselaers *et al*. [48] for complete code list | National Patient Register (1980-2008) |
| Parental separation/single-parent household | Having separated parents or living in a single-parent household | N/A | LISA^1^ (1990-2008) |
| Household living on public assistance | Public assistance during at least one year, where more than 50 percent of the yearly income constituted public assistance | N/A | LISA^1^ (1990-2008) |
| Residential instability | Three or more changes in place of residence | N/A | Total Population Register (1985-2008) |

^1^ Longitudinal Integration Database for Health Insurance and Labor Market Studies

***Supplementary Table 2.*** *Additional cohort characteristics, by exposure to childhood adversity, in employed individuals aged 19-29 years old residing in Sweden in 2009.*

|  | **All** | **No childhood adversity** | **Parental death** | **Parental mental disorder** | **Parental somatic disorder** | **Parental separation or single-parent household** | **Household public assistance** | **Residential instability** |  | **Total number of adversities** | | |
| --- | --- | --- | --- | --- | --- | --- | --- | --- | --- | --- | --- | --- |
|  |  |  |  |  |  |  |  |  |  | 1 | 2 | 3+ |
| All, (n, row percent) | 544,003 (100) | 294,808 (54) | 5,539 (1) | 40,284 (7) | 66,558 (12) | 201,148 (37) | 18,179 (3) | 20,173 (4) |  | 171,909 (32) | 55,995 (10) | 21,291 (4) |
| ***Sociodemographic factors^1^*** |  |  |  |  |  |  |  |  |  |  |  |  |
| *Parental country of birth* |  |  |  |  |  |  |  |  |  |  |  |  |
| Sweden | 467,181 (86) | 262,223 (89) | 4,586 (83) | 32,316 (80) | 55,382 (83) | 164,314 (82) | 11,701 (64) | 15,498 (77) |  | 145,158 (84) | 43,778 (78) | 16,022 (75) |
| Mixed | 50,557 (9) | 21,340 (7) | 656 (12) | 5,228 (13) | 7,026 (11) | 24,963 (12) | 3,446 (19) | 3,094 (15) |  | 3,141 (2) | 1,477 (3) | 491 (2) |
| Other Nordic | 9,250 (2) | 3,637 (1) | 140 (3) | 1,267 (3) | 1,517 (2) | 4,676 (2) | 793 (4) | 622 (3) |  | 18,120 (11) | 7,722 (14) | 3,375 (16) |
| EU25 (except Denmark, Finland and Sweden) | 10,369 (2) | 5,260 (2) | 92 (2) | 907 (2) | 1,540 (2) | 3,797 (2) | 900 (5) | 406 (2) |  | 2,285 (1) | 1,406 (3) | 607 (3) |
| The rest of the world | 6,646 (1) | 2,348 (1) | 65 (1) | 566 (1) | 1,093 (2) | 3,398 (2) | 1,339 (7) | 553 (3) |  | 3,205 (2) | 1,612 (3) | 796 (4) |
| *Type of residential area* |  |  |  |  |  |  |  |  |  |  |  |  |
| Big city area | 209,386 (38) | 107,983 (37) | 2,215 (40) | 15,789 (39) | 26,082 (39) | 83,294 (41) | 8,974 (49) | 8,558 (42) |  | 68,576 (40) | 23,838 (43) | 8,989 (42) |
| Intermediate (>90,000 inhabitants) | 196,807 (36) | 108,857 (37) | 1,941 (35) | 14,525 (36) | 23,723 (36) | 70,313 (35) | 5,926 (33) | 7,522 (37) |  | 60,938 (35) | 19,479 (35) | 7,533 (35) |
| Small (rural municipalities) | 137,810 (25) | 77,968 (26) | 1,383 (25) | 9,970 (25) | 16,753 (25) | 47,541 (24) | 3,279 (18) | 4,093 (20) |  | 42,395 (25) | 12,678 (23) | 4,769 (22) |
| *Family situation* |  |  |  |  |  |  |  |  |  |  |  |  |
| Married/living with partner  without children^2^ | 14,907 (3) | 9,204 (3) | 159 (3) | 910 (2) | 1,996 (3) | 4,033 (2) | 401 (2) | 543 (3) |  | 3,953 (2) | 1,249 (2) | 501 (2) |
| Married/living with partner  with children^2^ | 80,289 (15) | 43,121 (15) | 898 (16) | 6,907 (17) | 10,739 (16) | 29,393 (15) | 2,847 (16) | 3,087 (15) |  | 24,792 (14) | 8,795 (16) | 3,581 (17) |
| Single/divorced/separated/  widowed without children^2^ | 393,294 (72) | 214,136 (73) | 4,069 (73) | 28,508 (71) | 47,811 (72) | 144,760 (72) | 12,603 (69) | 14,372 (71) |  | 124,116 (72) | 40,003 (71) | 15,039 (71) |
| Single/divorced/separated/  widowed with children^2^ | 7,847 (1) | 2,973 (1) | 113 (2) | 1,061 (3) | 1,123 (2) | 4,239 (2) | 589 (3) | 559 (3) |  | 2,917 (2) | 1,269 (2) | 688 (3) |
| Children (≤20 years old)^2^ | 47,666 (9) | 2,5374 (9) | 300 (5) | 2,898 (7) | 4,889 (7) | 18,723 (9) | 1,739 (10) | 1,612 (8) |  | 16,131 (9) | 4,679 (8) | 1,482 (7) |

^1^ In 2009 ^2^ Living at home

***Supplementary Table 3.*** *Relative risks (RR) with 95% confidence intervals (CI) for associations between childhood adversity (CA), occupational class, and common mental disorders (CMDs) in employees in Sweden, aged 19-29 years old residing in Sweden in 2009. Included are also 47,493 individuals with a history of CMDs.*

|  | **Model 1^a^** | | **Model 2^b^** | | |
| --- | --- | --- | --- | --- | --- |
| **Childhood adversity** | **Non-manual workers** | **Manual workers** | **Non-manual workers** | **Manual workers** |  |
| **All** | 1 (REF) | 1.22 (1.20-1.24) | 1 (REF) | 1.19 (1.17-1.21) |  |
|  |  |  |  |  |  |
| Parental death |  |  |  |  |  |
| No | 1 (REF) | 1.22 (1.20-1.23) | 1 (REF) | 1.19 (1.17-1.21) |  |
| Yes | 1.12 (1.00-1.25) | 1.44 (1.37-1.53) | 1.10 (0.99-1.23) | 1.33 (1.26-1.40) |  |
| Parental mental disorder |  |  |  |  |  |
| No | 1 (REF) | 1.20 (1.19-1.22) | 1 (REF) | 1.19 (1.17-1.20) |  |
| Yes | 1.52 (1.46-1.58) | 1.82 (1.78-1.86) | 1.43 (1.38-1.49) | 1.63 (1.59-1.67) |  |
| Parental somatic disorder |  |  |  |  |  |
| No | 1 (REF) | 1.21 (1.19-1.23) | 1 (REF) | 1.18 (1.16-1.20) |  |
| Yes | 1.08 (1.04-1.12) | 1.37 (1.34-1.40) | 1.06 (1.03-1.10) | 1.29 (1.26-1.32) |  |
| Parental separation or single-parent household | |  |  |  |  |
| No | 1 (REF) | 1.16 (1.15-1.18) | 1 (REF) | 1.18 (1.16-1.21) |  |
| Yes | 1.35 (1.31-1.38) | 1.60 (1.57-1.63) | 1.28 (1.25-1.31) | 1.49 (1.46-1.52) |  |
| Household public assistance | |  |  |  |  |
| No | 1 (REF) | 1.21 (1.19-1.22) | 1 (REF) | 1.19 (1.17-1.21) |  |
| Yes | 1.49 (1.39-1.60) | 1.75 (1.71-1.80) | 1.34 (1.26-1.44) | 1.49 (1.45-1.55) |  |
| Residential instability |  |  |  |  |  |
| No | 1 (REF) | 1.21 (1.19-1.22) | 1 (REF) | 1.19 (1.17-1.20) |  |
| Yes | 1.34 (1.26-1.42) | 1.72 (1.68-1.77) | 1.26 (1.18-1.34) | 1.50 (1.45-1.54) |  |

^a^ Model 1: Crude ^b^ Model 2: Adjusted for age, sex, education, family situation, parental country of birth, residential area, LTSA and DP in 2009 and somatic morbidity

***Supplementary table 4.*** *Associations between cumulative childhood adversity (CA), occupational class, and common mental disorders (CMDs) in employees in Sweden, aged 19-29 years old residing in Sweden in 2009. Relative risks (RRs) with 95% confidence intervals (CIs). Included are also 47,493 individuals with a history of CMDs.*

|  | **Model 1^a^** | | **Model 2^b^** | |
| --- | --- | --- | --- | --- |
| **Total number of childhood adversities** | **Non-manual workers** | **Manual workers** | **Non-manual workers** | **Manual workers** |
| 0 | 1 (REF) | 1.15 (1.12-1.17) | 1 (REF) | 1.17 (1.15-1.19) |
| 1 | 1.23 (1.20-1.26) | 1.46 (1.43-1.49) | 1.19 (1.16-1.22) | 1.41 (1.38-1.44) |
| 2 | 1.49 (1.44-1.55) | 1.78 (1.74-1.81) | 1.41 (1.36-1.46) | 1.62 (1.58-1.66) |
| 3+ | 1.63 (1.54-1.73) | 2.01 (1.96-2.07) | 1.50 (1.42-1.59) | 1.76 (1.71-1.81) |

^a^ Model 1: Crude ^b^ Model 2: Adjusted for age, sex, education, family situation, parental country of birth, residential area, LTSA and DP in 2009 and somatic morbidity
